# Towards an EKG for SBO: A Neural Network for Detection and Characterization of Bowel Obstruction on CT

**DOI:** 10.1007/s10278-024-01023-y

**Published:** 2024-02-22

**Authors:** Paul M. Murphy

**Affiliations:** University of California-San Diego, UCSD Radiology, 9500 Gilman Dr, La Jolla, 200 W Arbor Dr, San Diego, CA 92103 USA

**Keywords:** Bowel obstruction, Neural networks, Embedding, Segmentation, Quantitative imaging

## Abstract

A neural network was developed to detect and characterize bowel obstruction, a common cause of acute abdominal pain. In this retrospective study, 202 CT scans of 165 patients with bowel obstruction from March to June 2022 were included and partitioned into training and test data sets. A multi-channel neural network was trained to segment the gastrointestinal tract, and to predict the diameter and the longitudinal position (“longitude”) along the gastrointestinal tract using a novel embedding. Its performance was compared to manual segmentations using the Dice score, and to manual measurements of the diameter and longitude using intraclass correlation coefficients (ICC). ROC curves as well as sensitivity and specificity were calculated for diameters above a clinical threshold for obstruction, and for longitudes corresponding to small bowel. In the test data set, Dice score for segmentation of the gastrointestinal tract was 78 ± 8%. ICC between measured and predicted diameters was 0.72, indicating moderate agreement. ICC between measured and predicted longitude was 0.85, indicating good agreement. AUROC was 0.90 for detection of dilated bowel, and was 0.95 and 0.90 for differentiation of the proximal and distal gastrointestinal tract respectively. Overall sensitivity and specificity for dilated small bowel were 0.83 and 0.90. Since obstruction is diagnosed based on the diameter and longitude of the bowel, this neural network and embedding may enable detection and characterization of this important disease on CT.

**Supplementary Information:**

The online version contains supplementary material available at 10.1007/s10278-024-01023-y.

## Introduction

Neural networks are revolutionizing the interpretation of medical imaging [1, 2]. Their strong performance has already had huge impacts in many areas, such as in classification of studies as normal or diseased, and in segmentation of structures such as organs or tumors [3]. Neural networks can also serve as “universal function approximators”[4], thus can represent functions beyond classification or segmentation. This usage may have additional impacts in the branch of radiology known as quantitative imaging, in which numeric features beyond those imaged directly by the modality are mapped across the same region of space [5]. These numeric features can help characterize diseases in ways beyond merely reporting their presence or location. This study investigates neural networks for quantitative imaging of bowel obstruction.

Bowel obstruction is an extremely common cause of acute abdominal pain, found in approximately 15% of all emergency department presentations for that chief complaint [6]. It often arises due to adhesions formed in the peritoneal cavity after surgery, but has many other causes such as neoplastic masses or inflammatory strictures [7]. These abnormalities preclude downstream passage of bowel contents. The upstream bowel becomes dilated in response. A diameter of 30 mm is commonly used as a clinical threshold for abnormal dilation of the small bowel [8]. A transition point in diameter along the longitudinal course of the bowel is often the location of the cause of the obstruction, and helps distinguish obstruction from ileus. Bowel obstruction is well depicted on multiple modalities, including CT, which is the focus of this work.

This study investigates neural networks as “universal function approximators” for two numeric features of bowel obstruction: the diameter of the bowel, and its longitudinal position along the course of the gastrointestinal tract (henceforth referred to as “longitude”). The predictions of a neural network are compared to manual segmentations and to manual measurements of diameter over a range of longitudes. These reference standards can be defined easily, though only approximately and incompletely, since the bowel is not a perfect cylinder, and since images contain more voxels than possible to annotate manually. Nonetheless, the common clinical usage of bowel diameter reflects its utility as an approximation, and measurements over a subset of query points may serve as an adequate sample.

With these numeric features, bowel obstruction can be detected using clinical thresholds for diameter, and can be characterized by plotting diameter against longitude to show transition points. The purpose of this study is to assess the performance of a neural network for bowel segmentation and for prediction of bowel diameter and longitude.

## Methods and Materials

### Study Design

This retrospective study was approved by the institutional review board with a waiver of informed consent due to its minimal risk. 202 CT scans from 165 patients were identified by searching a radiology report database from March to June 2022 for impressions containing the phrase “bowel obstruction” whether prefaced by the word “small”, “large”, or “no”. These criteria were intended to include scans with the presence and absence of bowel obstruction and with a wide range of bowel diameters. Patients from the emergent, inpatient, and outpatient contexts were all included. Scans with intravenous contrast, enteric contrast, and no contrast, from a variety of modality vendors at the author’s institution were all included.

Scans were excluded if they did not include the entire abdomen and pelvis, or if the patient was not in the supine position. Scans were not excluded due to poor image quality, since generalization over all levels of image quality was desired. Subjects were partitioned randomly into training and test datasets. All scans from a given subject were included in the same dataset. Demographic and technical information for the included subjects and scans in each dataset is given in Table 1. All protected health information was removed prior to analysis to ensure HIPAA compliance.

**Table 1:** Demographics of training and test data sets. Proportions of clinical features are reported for all subjects. Maximum age greater than 89 is reported as 90 + per HIPAA guidelines. Patient classes E, I, and O, correspond to emergency department patients, inpatients, and outpatients respectively. Averages and ranges are reported for technical features for all scans

	**Training**	**Test**
***Sample size***		
* Subjects*	50	115
* Scans*	60	142
* Scans per subject*	1.3 (1–3)	1.2 (1–4)
***Demographics***		
* Sex (F)*	0.48 (24/50)	0.51 (59/115)
* Age (yrs)*	F:60, M:59	F:61, M:59
* Age range (yrs)*	19–90 +	28–90 +
* Patient class per scan*	E: 0.67 (40/60) I: 0.17 (10/60) O: 0.17 (10/60)	E: 0.63 (88/142) I: 0.26 (36/142) O: 0.11 (16/142)
***CT Technique***		
* Intravenous contrast*	0.87 (52/60)	0.81 (116/142)
* Enteric contrast*	0.13 (8/60)	0.06 (8/142)
* Noncontrast*	0.10 (6/60)	0.16 (23/142)
***Scan Dimensions – interpolated***		
* Matrix size*	512 × 512	512 × 512
* Number of axial slices*	526 (500–726)	529 (483–723)
* Slice thickness (mm)*	0.90 (0.61–1.20)	0.90 (0.68–1.19)
* In-plane voxel size (mm)*	0.75 (0.58–0.98)	0.78 (0.58–0.98)
***Scan Dimensions – resized***		
* Matrix size*	256 × 256	256 × 256
* Number of axial slices*	192	192
* Slice thickness (mm)*	2.77 (2.12–3.60)	2.80 (2.20–3.58)
* In-plane voxel size (mm)*	1.51 (1.17–1.95)	1.56 (1.16–1.95)
***Query points***		
* Per scan*	18.4	18.1
* Total*	1105	2569

Including multiple scans from some subjects did result in overrepresentation of those subjects; however, it was necessary to ensure inclusion of clinical and technical variations. When patients are scanned more than once, the intent is often to evaluate for clinical changes, such worsening or improvement of obstruction, or with a different diagnostic technique, such as intravenous or enteric contrast. Including these scans was important to ensure generalization, albeit at a cost of overrepresentation. The overall impact was likely not large since the maximum number of scans for a single patient was 3 in the training data set and 4 in the test data set. Since the training and test data sets were partitioned by subject rather than scan, memorization of the training data set did not impact assessment of the test data set.

### Data Annotation, Embedding, Augmentation, and Normalization

Scans in the training data set were annotated using a Tobii 4c eye tracking device and a custom extension in 3D Slicer rather than vendor’s analysis software, as described previously [9–11]. Briefly, the eye tracker was used to record locations within the imaging volume while a radiologist cast their gaze at the centerline of the bowel. The display size of the imaging volume on the screen varied, since the radiologist could freely zoom, pan, and scroll during recording. The diameter of a superimposed ROI was manually adjusted to match the approximate diameter of the bowel for all locations along the centerline of the gastrointestinal tract in real time during recording at a nominal rate of 60 Hz.

Gaze locations were aggregated over approximately 10 s time intervals of recording, into one or more segments for each organ (esophagus, stomach, duodenum, etc.). Each segment was comprised of a series of gaze points along the centerline of the bowel, with a corresponding diameter for each point, and an organ to which each segment belonged. These collections of information are subsequently referred to as “visual annotations”. All annotations were performed by one radiologist with 12 years of experience.

Each organ was assigned an initial longitude (10, 20, 30, etc., as in Table 2). The longitude along each segment was adjusted proportionally to the total length of the organ, according to the formula below.

**Table 2:** Longitudes of the gastrointestinal tract

		**Organ**	**Longitude**		
Esophagus	10	Ileum	60	Transverse Colon	110
Stomach	20	Terminal Ileum	70	Descending Colon	120
Duodenum	30	Appendix	80	Sigmoid Colon	130
Treitz Jejunum	40	Cecum	90	Rectum	140
Jejunum	50	Ascending Colon	100	Anus	150

$$\begin{aligned}&Longitude=Initial\;Longitude+\\&\left(Final Longitude-Initial\;Longitude\right)*\frac{Length}{Total\;Length}\end{aligned}$$

For instance, if the stomach was 25 cm in total length, and the gastric fundus was 5 cm from the beginning, it would be assigned a longitude of 22. Likewise, if the gastric antrum were 5 cm from the end, it would be assigned a longitude of 28. Since the small bowel was typically comprised of multiple segments, an order that minimized the distance between segments was identified and used to calculate longitude. Whereas multipart segmentations of prior studies represent different organs as a categorical variable [11], longitude represents the entire gastrointestinal tract as a continuous variable.

These specific definitions of longitude were intended to reflect anatomic boundaries that could be located with high confidence, such as the gastroesophageal junction, pylorus, ligament of Treitz, ileocecal valve, splenic, hepatic, and sigmoid flexures, anterior peritoneal reflection, and anal sphincters. The small bowel was further divided into proximal jejunum (“Treitz jejunum”), mid jejunum, mid ileum, and terminal ileum. Boundaries between these segments of the small bowel are only approximate, but differentiation is important for characterization of obstruction. In addition, some segments of the gastrointestinal tract may be continuous in longitude but discontiguous in space, for instance the terminal ileum and appendix, or an end colostomy and Hartman’s pouch status post colectomy. This does not limit annotation, but must be considered during interpretation of longitude.

**Fig. 1:**
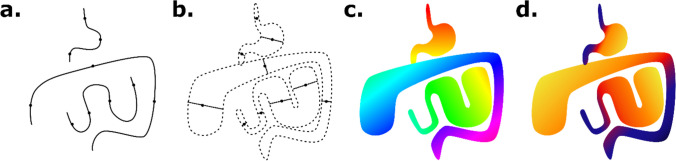
Embedding of longitude and diameter. Gaze points along the centerline of the bowel were recorded with an eye tracking device (**a**). The bowel diameter was recorded for each gaze point, and used to segment the region of space around the centerline (**b**). The longitude was calculated for each voxel based on the distance along the gastrointestinal tract (**c**). The diameter was calculated for each voxel based on the diameter of the closest gaze points (**d**)

These visual annotations were embedded into a binary segmentation volume, in which the value of each voxel was set to one if the distance between voxel and closest segment was within the radius of the segment, and zero elsewhere. The diameter and longitude were embedded into volumes similarly, with the value of each voxel set to that of the closest segment if within its radius. Since diameter and longitude had only been recorded for the gaze points that define the beginning and end of each segment, their value at each voxel was interpolated based on the fractional distance of the projection of the voxel along the segment. A diagram of the visual annotation and embedding process is shown in Fig. 1.

Only thick slice multiplanar reconstructions were available for CT scans over the period included in this study. To permit augmentation, near-isotropic volumes were reconstituted by interpolation and summation of the thick slice axial, sagittal, and coronal series. Scan dimensions after interpolation from thick slice to near-isotropic volumes are given in Table 1. Data augmentation of the near-isotropic volumes was performed by random rotation, translation, and scaling by up to 15 degrees, 15 mm, or 15% respectively. A total of 14 different transformations were applied to each annotated scan to generate the augmented training data set, with the number of transformations chosen so as to fill the entirety of the 128 GB of system memory on the computer used for training. CT scans were window/leveled using values of 400/40 HU. Diameters were scaled by the voxel dimensions before training and after prediction.

All volumes were resized to 256 × 256 × 192 voxels prior to being used in the neural network. Since the initial number of axial slices varied, scans were “zero”-padded to the next greatest multiple of 192 axial slices prior to resizing, to avoid interpolation at this step. A padding value of -1000 HU rather than zero was actually used to emulate surrounding air. This padding added variation to voxel sizes during resizing, which may be a source of error, but was necessary to ensure entire scans could be included.

### Neural Network

**Fig. 2:**
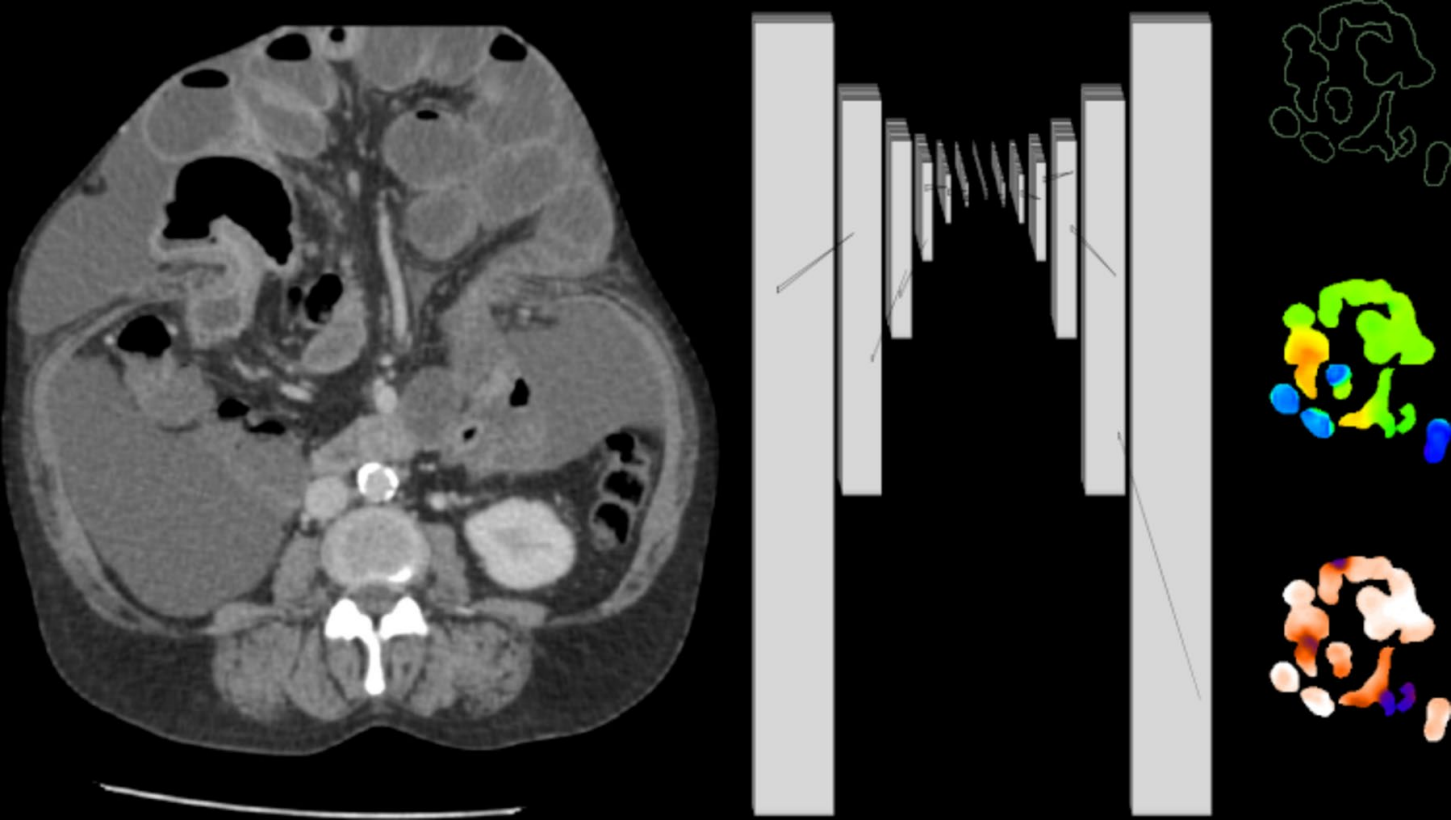
Neural network diagram. A 3d U-net convolutional neural network was implemented, with the CT scan volume as the single channel of input, and segmentation, longitude, and diameter maps as three channels of output

A 3D U-net convolutional neural network [12, 13] was implemented and trained using Keras and Tensorflow version 2.12 [14, 15]. The single channel of input to the model was the entire CT scan. The three channels of output of the model were segmentation, diameter, and longitude volumes, as shown in the neural network diagram (Fig. 2) with specific parameters listed in Table 3. The model had 37 M parameters in total and was trained starting from random parameters on an NVIDIA A6000 GPU. Binary crossentropy loss was used for segmentation, and mean squared error losses were used for diameter and longitude. Each loss was weighted to approximately the same order of magnitude to ensure all were optimized during training. The neural network parameters were optimized using the Adam optimizer with a learning rate of 1e-3 for 222 epochs, exclusively on the training data set. To monitor optimization performance, 5% of the augmented training data set was reserved for validation. The test data set was not used for either training or validation. The model and training were otherwise similar to prior [11].

**Table 3:** Neural network parameters. The shape and number of channels are listed for each of the layers of the neural network. Each intermediate layer was composed of two convolutions, batch normalization, activation, dropout, and either maxpooling or upsampling layers. Convolution filter size was 3, dropout rate was 0.1, and maxpooling or upsampling ratio was 2. Feed forward connections were added as concatenations between intermediate layers of the same shape. The three output layers are connected in parallel to the subsequent intermediate layer but not to each other

Layer	Shape	Channels
Input (CT)	256 × 256 × 192	1
1	256 × 256 × 192	16
2	128 × 128 × 96	32
3	64 × 64 × 48	64
4	32 × 32 × 24	128
5	16 × 16 × 12	256
6	8 × 8 × 6	512
7	4 × 4 × 3	512
8	8 × 8 × 6	256
9	16 × 16 × 12	128
10	32 × 32 × 24	64
11	64 × 64 × 48	32
12	128 × 128 × 96	16
Output (seg.)	256 × 256 × 192	1
Output (long.)	256 × 256 × 192	1
Output (diam.)	256 × 256 × 192	1

**Fig. 3:**
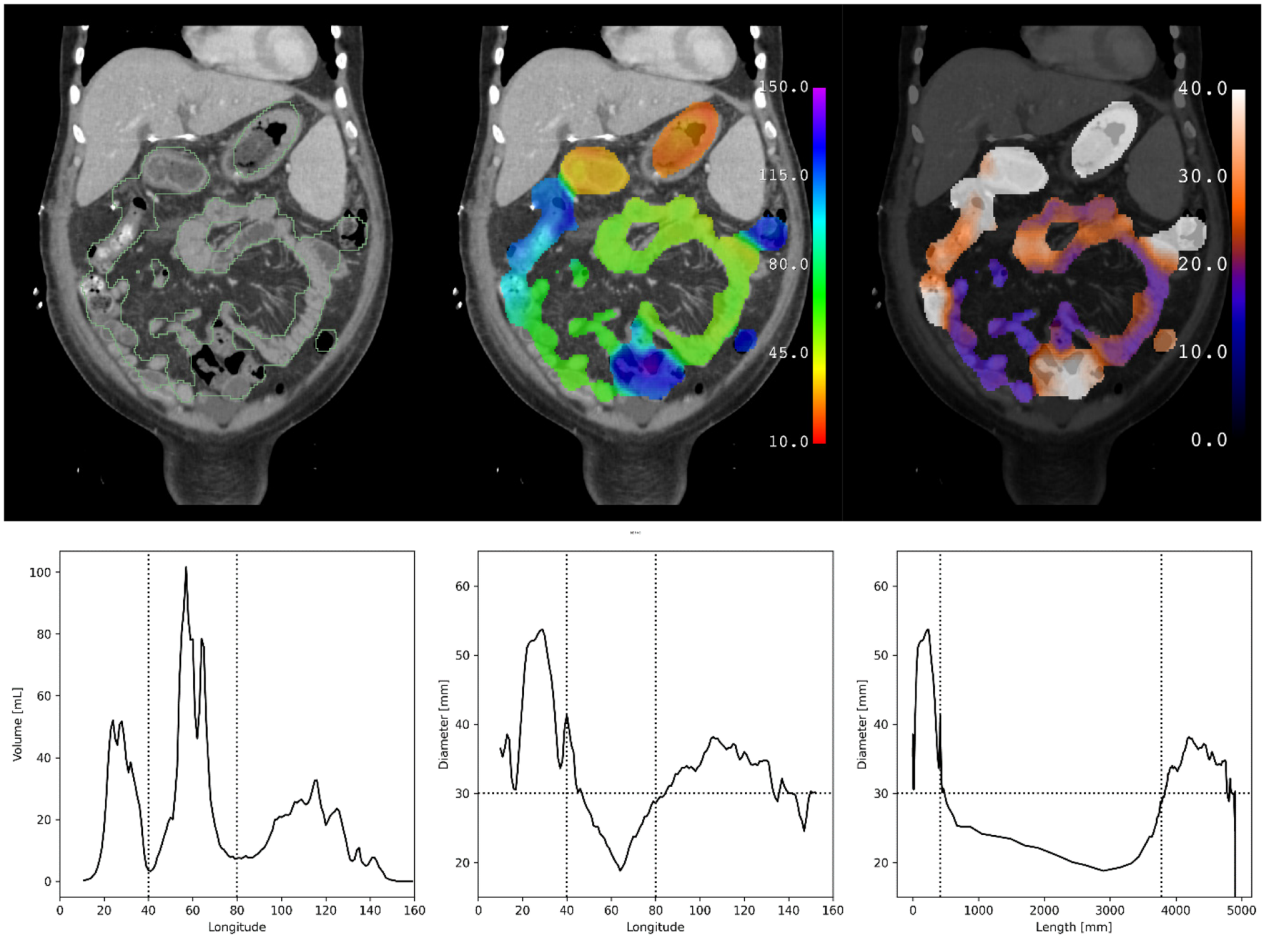
Test subject with no bowel obstruction. Segmentation (green outline), longitude (rainbow), and diameter (hot metal blue) predictions superimposed on a coronal slice of a CT scan of a subject from the test data set with no bowel obstruction. Graphical representations of volume and diameter (averaged over 1 unit intervals of longitude) versus longitude and length are shown for the same subject. Vertical dotted lines indicate the ligament of Treitz and ileocecal valve. Horizontal dotted line indicates the threshold for small bowel obstruction. No small bowel dilation and no transition point are present

**Fig. 4:**
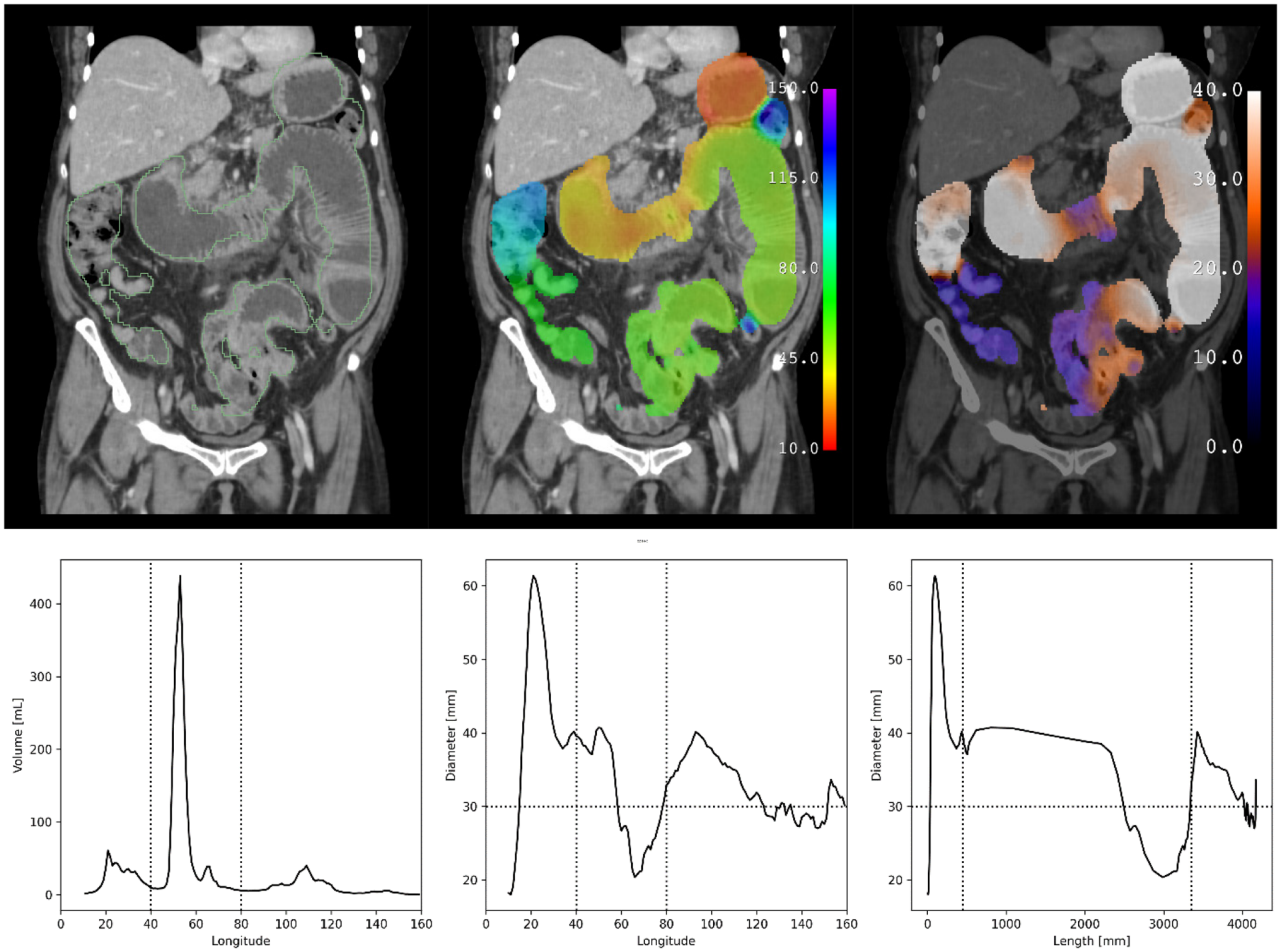
Test subject with bowel obstruction. Segmentation (green outline), longitude (rainbow), and diameter (hot metal blue) predictions superimposed on a coronal slice of a CT scan of a subject from the test data set with bowel obstruction. Graphical representations of volume and diameter (averaged over 1 unit intervals of longitude) versus longitude and length are shown for the same subject. Vertical dotted lines indicate the ligament of Treitz and ileocecal valve. Horizontal dotted line indicates the threshold for small bowel obstruction. Small bowel dilation and a transition point are present are present in this subject, as seen in the graph of diameter versus length. Several loops of nondilated distal ileum are noted to be erroneously excluded from the segmentation

**Fig. 5:**
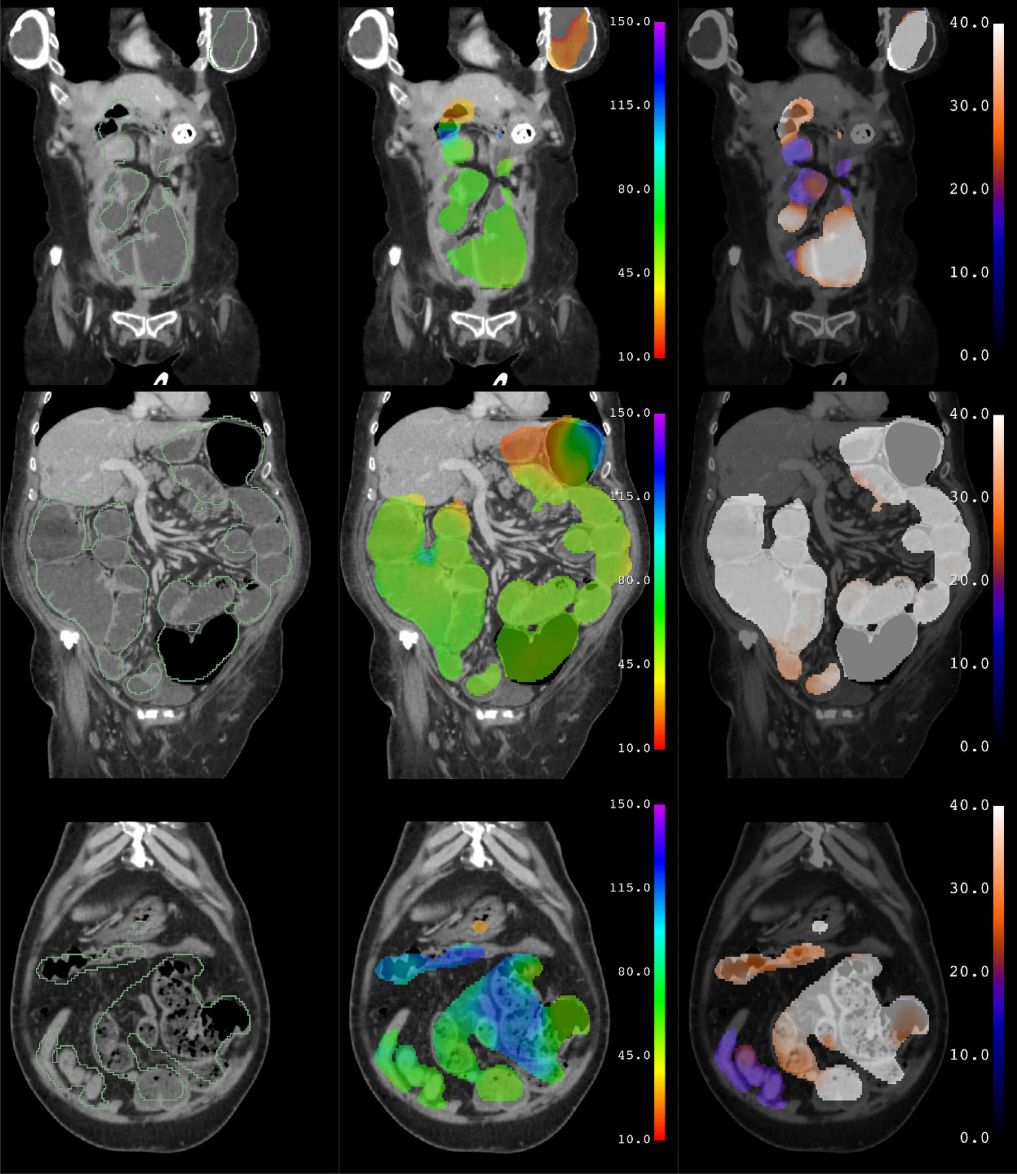
Test subjects with erroneous predictions. Coronal slices illustrating errors associated with abnormalities such as a gastrostomy tube and breast implants (top), liquid colonic contents (middle), and fecalized small bowel contents (bottom). In the first subject (top), the left breast implant is erroneously segmented and assigned a longitude corresponding to stomach, while the actual stomach containing a percutaneous gastrostomy tube is not included in the segmentation. In the second subject (middle), the colon contains fluid due to a diarrheal state, rather than feces as usual, and is erroneously assigned a longitude corresponding to small bowel. In the third subject (bottom), the small bowel contains fecalized contents due to delayed transit, rather than liquid as usual, and is erroneously assigned a longitude corresponding to colon

**Fig. 6:**
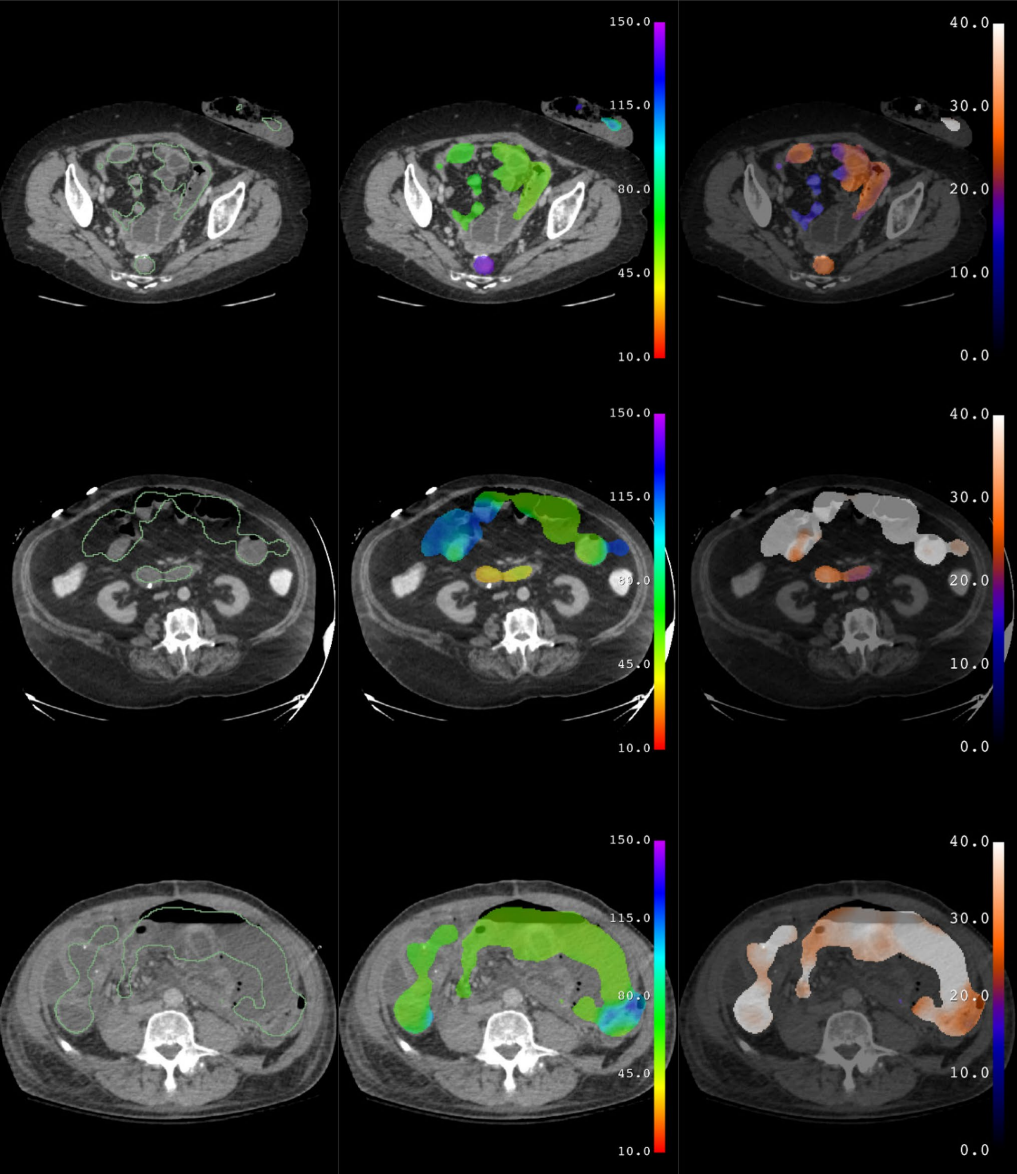
Test subjects with erroneous predictions. Axial slices illustrating errors associated with abnormalities such as a colostomy and Hartman’s pouch (top), enteric contrast within the colon (middle), and ascites with pneumoperitoneum (bottom). In the first subject (top), the ostomy bag is erroneously segmented and assigned a longitude corresponding to colon, while the small bowel near the Hartman’s pouch is excluded from the segmentation. In the second subject (middle), the colon contains enteric contrast, rather than feces as usual, and is erroneously excluded from the segmentation. In the third subject (bottom), ascites and pneumoperitoneum outside of the bowel are erroneously included in the segmentation

Predictions of the trained model are shown for subjects from the test data set with no bowel obstruction (Fig. 3) and with bowel obstruction (Fig. 4). Erroneous predictions and associated abnormalities in subjects from the test data set are shown as well (Figs. 5, 6). Predicted binary segmentation masks are applied to both the diameter and longitude volumes since their support may differ.

### Graphical Representations

Graphs plotting volume and diameter versus longitude are shown alongside corresponding model predictions in Figs. 3 and 4 calculated over 1 unit intervals of longitude. Assuming a cylindrical shape, length can be calculated for any interval of longitude using the formula below in which L is length, V is volume, and D is diameter.

$$L=\frac{4}{\pi } \frac{V}{{D}^{2}}$$

A graph plotting diameter versus length rather than longitude is shown after this change of variables. The accuracy of these lengths was not in the scope of this study to assess, though they are of the same order of magnitude as reported lengths of the human gastrointestinal tract [16].

### Reference Standards

To evaluate the predictions of the model, manual annotation was performed. All manual annotations were performed in 3D Slicer [9] by a radiologist with 12 years of experience.

Three slices of each CT scan were randomly selected for manual segmentation. It was time-prohibitive to perform manual segmentations of every slice of every CT scan, since there were hundreds of scans with several hundred slices apiece. Instead, three slices were chosen under the constraint that the first included the stomach, the second included the small bowel, and the third included the colon, to ensure even sampling. All parts of the gastrointestinal tract were manually segmented on each of these three slices. Subjects who had more than one scan were slightly overrepresented since this analysis was performed across scans.

Multiple query points along the centerline of the gastrointestinal tract were randomly selected for diameter measurement. It was also time-prohibitive to perform diameter measurements over every voxel within the CT scans. Instead, one query point was chosen for each of the fifteen parts of the gastrointestinal tract listed in Table 2. Four query points were chosen for the jejunum and ileum, including the segment of jejunum adjacent to the ligament of Treitz and including the terminal ileum, due to their length and importance in bowel obstruction. The diameter was manually measured as the distance between the outer margins of the bowel in the short axis at this point.﻿ 

The longitude of each query point could not be measured with complete accuracy, so was presumed to correspond to the midpoint of each part of the gastrointestinal tract. For instance, the longitude for a query point in the stomach was always presumed to be 25. Subjects who had bowel resections at which measurements could not be performed were slightly underrepresented since this analysis was performed across query points.

The manual annotations used for testing took less time to perform than the visual annotations used for training; thus, it was possible to create a relatively larger test data set, which was useful to assess generalization of the neural network’s predictions.

### Statistical Analysis

**Fig. 7:**
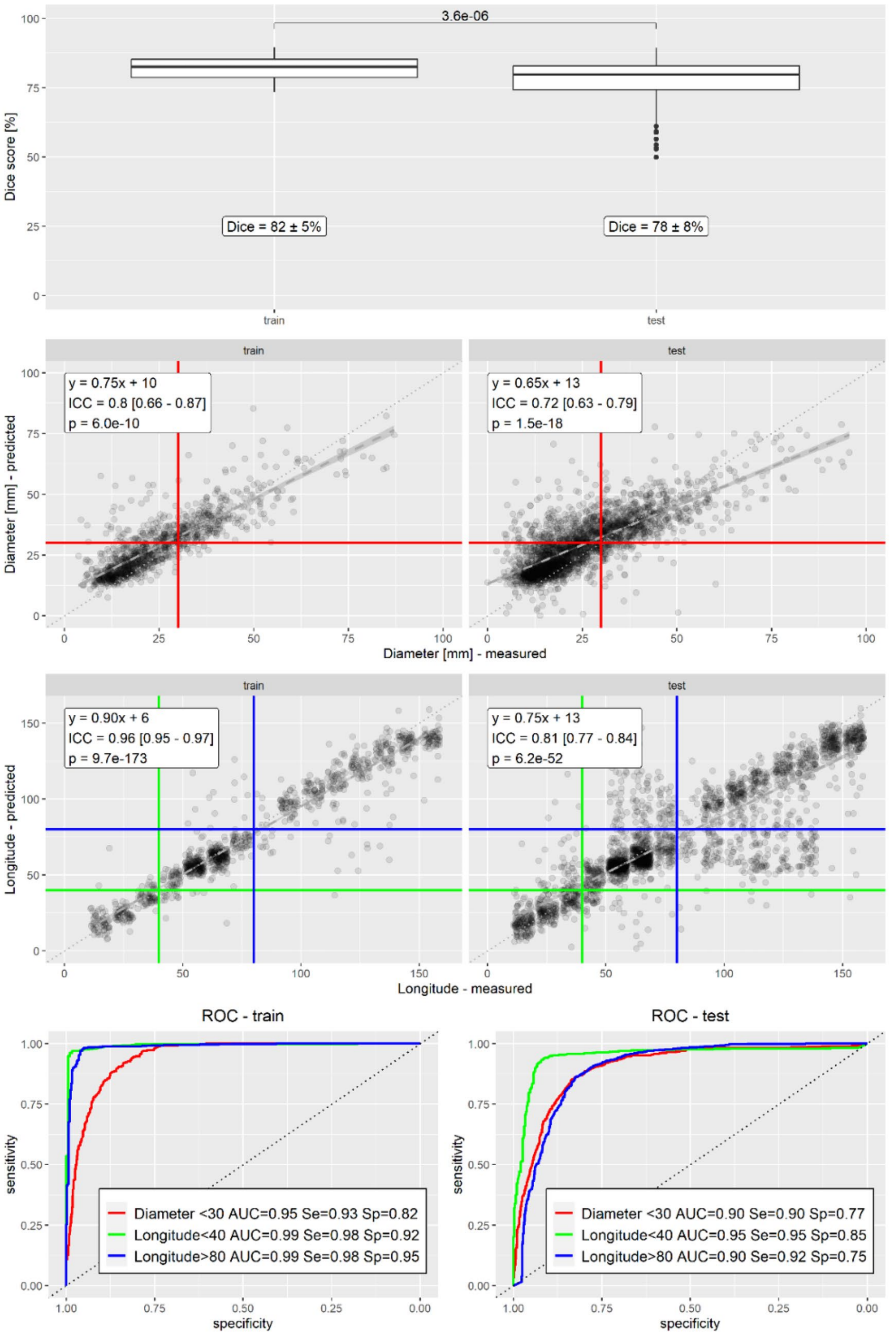
Statistical analysis. Dice scores were calculated between manual and predicted segmentations. The p-value of a t-test between Dice scores of the training and test data sets is also shown. Intraclass correlations were calculated between manual and predicted values for diameters and longitudes. The equation of the best linear fit and thresholds used for ROC analysis are also shown. The longitude scatterplot is made with jitter to avoid superimposition of presumed longitudes. ROC curves and AUROCs were calculated using thresholds of 30 mm for diameter, and 40 and 80 for longitude. Sensitivity and specificity are shown using the same thresholds for predictions as measurements. Each statistic was calculated for the training and test data sets separately

Dice scores were calculated to assess agreement between manual segmentations and segmentations predicted by the model [17], and were compared using the unpaired t-test. Intraclass correlation coefficients (ICC, two way, agreement) were calculated to assess agreement between measurements at query points and the values predicted by the model for both diameter and longitude [18, 19], and were compared using the F-test. Receiver operating curves were calculated for diameter, using a threshold of 30 mm corresponding to the clinical definition of abnormally dilated small bowel, and for longitude, using thresholds of 40 corresponding to the ligament of Treitz and 80 corresponding to ileocecal valve, between which lies the small bowel. AUROCs between training and test data sets were compared with Delong’s test. Results are shown in Fig. 7. Similar analyses are shown for subsets of the gastrointestinal tract in Supplemental Figures S1, S2, and S3.

To evaluate the joint performance of all three predictions, sensitivity and specificity were calculated for query points corresponding to dilated small bowel. Predictions were considered positive if the query point was included in the binary segmentation, had a diameter greater than 30 mm, and had a longitude between 40 and 80. Sensitivity and specificity for nondilated small bowel was also calculated. McNemar’s test was calculated to assess discordance. ROC analysis could not be performed for joint predictions, since more than one parameter must be varied.

Comparisons with manual annotations are reported for training as well as test data sets, since the model was trained on visual annotations but not on manual annotations. All statistical analysis was performed in R using the ISS and pROC packages [20].

## Results

### Demographics

Demographics and technical features of subjects and scans included in the training and test data sets are reported in Table 1. The number and proportion of query points at which measured and predicted values met thresholds for diameter and longitude are reported in Table 4.

**Table 4:** Query Points. Proportion (and number) of query points in the training and test data sets meeting thresholds for diameter and longitude. “Measured” indicates that the manual measurement of diameter and longitude at that query point met the threshold. “Predicted” indicates that the model prediction at that query point met the threshold

Threshold	Type	**Training**	**Test**
*Number of Query Points*		1105	2569
*Diameter* > *30 mm*	*Measured*	0.29 (323)	0.31 (800)
	*Predicted*	0.40 (446)	0.44 (1136)
*Longitude* < *40*	*Measured*	0.16 (186)	0.16 (420)
	*Predicted*	0.17 (192)	0.18 (459)
*Longitude* > *80*	*Measured*	0.32 (423)	0.40 (1015)
	*Predicted*	0.38 (415)	0.34 (882)
*Dilated Small Bowel*	*Measured*	0.11 (120)	0.10 (267)
	*Predicted*	0.13 (148)	0.18 (450)
*Nondilated Small Bowel*	*Measured*	0.34 (376)	0.34 (867)
	*Predicted*	0.29 (316)	0.23 (597)

### Segmentation

Dice scores between model predictions and manual segmentations were calculated over all scans in the training and test data sets (n = 60 and n = 142), as shown in Fig. 7 (top). The Dice scores were 82 ± 5% and 78 ± 8% for the training and test data sets. An unpaired t-test indicated that the that the decrease in Dice scores between training and test data sets was statistically significant (p = 3.6e-6).

### Diameter

Intraclass correlation coefficients between model predictions and manual measurements of diameter were calculated over all query points along the gastrointestinal tract in the training and test data sets (n = 1105 and n = 2569), as shown in Fig. 7 (middle). The intraclass correlation coefficients were 0.8 and 0.72 for the training and test data sets. An F-test indicated both were statistically significantly different than zero (p = 6.0e-10 and p = 1.5e-18). This represents moderate agreement for the test data set, using standard thresholds for agreement [19].

An ROC curve for diameter was calculated over these same query points (n = 1105 and n = 2569), as shown in Fig. 7 (bottom). The proportion of query points at which measured and predicted diameter was above 30 mm for the training and test data sets are given in Table 4. The AUROC for diameter above 30 mm was 0.95 and 0.90 for the training and test data sets. DeLong’s test indicated that the decrease in AUROC between training and test data was statistically significant for diameter (p = 2.1e-6).

### Longitude

Intraclass correlation coefficients between model predictions and manual measurements of longitude were calculated over all query points along the gastrointestinal tract in the training and test data sets (n = 1105 and n = 2569), as shown in Fig. 7 (middle). The intraclass correlation coefficients s were 0.85 and 0.85 for the training and test data sets. An F-test indicated both were statistically significantly different than zero (p = 9.7e-173 and p = 6.2e-52). This represents good agreement for the test data set.

ROC curves for longitude were calculated over the same query points (n = 1105 and n = 2569), as shown in Fig. 7 (bottom). The proportion of query points at which measured and predicted longitude was below 40 or above 80 for the training and test data sets are given in Table 4. The AUROC for prediction of longitude below 40 was 0.99 and 0.95 in the training and test data sets. The AUROC for prediction of longitude above 80 was 0.99 and 0.90 in the training and test data sets. DeLong’s test indicated that the decrease in AUROC between training and test data was statistically significant both for both longitude thresholds (p = 2.9e-11 and p = 2.2e-16).

### Joint Performance

Joint performance of both diameter and longitude was assessed using thresholds that represented dilated small bowel and nondilated small bowel. The proportion of query points meeting these thresholds is given in Table 4. Sensitivity and specificity of predictions for measurements were calculated over all query points in the training and test data sets.

Dilated small bowel was defined as diameter above 30 mm and longitude between 40 and 80. Sensitivity and specificity for dilated small bowel were 0.90 and 0.96 in the training data set, and 0.83 and 0.90 in the test data set.

Nondilated small bowel was defined as diameter below 30 mm and longitude between 40 and 80. Sensitivity and specificity for nondilated small bowel were 0.82 and 0.90 in the training data set, and 0.54 and 0.92 in the test data set, reflecting relative insensitivity of segmentation of smaller structures.

McNemar’s test indicated that the discordance between measurements and predictions was statistically significant both for dilated and nondilated small bowel in the training and test data sets (p < 2.2e-16).

## Discussion

### Summary

These results demonstrate moderate-to-good agreement and strong diagnostic performance for features of bowel obstruction, despite lower performance for segmentation. Automated detection of acute abnormalities for patient triage is an established use case for artificial intelligence in radiology [1, 2]. These results may add small bowel obstruction to the list of abnormalities amenable to automated detection.

In addition, characterization of quantitative imaging features may bolster the explainability of model predictions beyond detection alone. Graphical representations can depict the relationship between longitude and diameter across the bowel, as an EKG does for time and voltage across the heart, so may serve analogously as an “EKG for SBO”. Such characterization may facilitate patient triage by quantifying the severity of obstruction based on the diameter and length of the involved segments.

This study also demonstrates that eye tracking can be used for development of artificial intelligence tools with clinical applications. Eye tracking enabled visual annotation of features that would be prohibitive to perform manually. As the accuracy and precision of eye tracking devices improve, more applications of these devices in radiology research and clinical practice may develop as well.

### Previous studies

Dice scores for segmentation of the gastrointestinal tract are similar to some but lower than other results reported previously [3, 21–27], which range from 0.60 to 0.95 over different modalities and parts of the gastrointestinal tract. This may represent a limitation of training with visual annotations exclusively, arising from the need to annotate diameter and longitude in addition to segmentation. Utilization of publicly available training data and models [3] for segmentation may improve performance in future efforts; however, the prevalence of bowel obstruction in those resources remains to be evaluated, and additional diameter and longitude annotations may still be required.

Intraclass correlation coefficients for diameter are similar to prior studies using this same training dataset (reference blinded for review), but ICC for longitude and AUROC for diameter and longitude were not reported previously. AUROC reported in this study are also similar to those reported in other studies that have used neural networks to detect bowel obstructions on radiographs and transition points on CT [28–31], which ranged from 0.84 to 0.97 though direct comparison is limited due to differences in modality and approach.

### Limitations

There are several limitations to this study.

First, diameter is widely used clinically [6, 8], but is not well-defined since the gastrointestinal tract is not a perfect cylinder. The short axis outer diameter was used as a reference standard in this study, but the long axis or inner diameters were not measured. Incorporating those features into future models will be the subject of future research. Any geometric model can only approximate the underlying structures. Nonetheless, diameter is relevant alongside segmentation volumes, since it differentiates dilated bowel from a cluster of nondilated bowel that may have the same volume even if segmented accurately. Concurrent diameter and volume predictions may also allow the calculation of bowel length, which will be the topic of future research.

Second, longitude is based on anatomic landmarks that can be easily identified on CT scans, such as the gastroesophageal junction or pylorus. However, few such anatomic landmarks exist, particularly for the small bowel between the ligament of Treitz and the ileocecal valve. This limits assessment of longitude, especially within the part of the gastrointestinal tract that is the longest and most important for bowel obstruction. Nonetheless, differentiation between jejunum and ileum by longitude predictions is appreciated in examples such as Fig. 4 and in Supplemental Fig. 7.

Lastly, the results of this study were evaluated over only a subset of slices and points within the CT scans. These slices were chosen randomly, and points were chosen randomly along the centerline of the gastrointestinal tract; thus, these results may generalize. It was time-prohibitive to assess performance outside this subset. Assessment was limited in other ways as well. Model predictions were not evaluated against clinical outcomes at the subject level, and it is uncertain whether the performance obtained in this study will suffice to be useful clinically. This was a single institution study including a limited number of subjects in both the training and test data sets, since an external validation set of patients with bowel obstruction was not available. These limitations will also be topics of future research.

## Conclusion

The results of this study demonstrate strong diagnostic performance of a neural network for features of bowel obstruction. The embedding of diameter and longitude may help characterize aspects of this disease beyond merely its presence or absence. Neural networks such as described here promise to revolutionize quantitative imaging of bowel obstruction, as they have other aspects of radiology.

### Supplementary Information

Below is the link to the electronic supplementary material.

Supplementary file1 (DOCX 1158 KB)

## Data Availability

Data that support the findings of this study are available from the author upon reasonable request with restrictions.
