# Supplementary material for: Towards an EKG for SBO: A Neural Network for Detection and Characterization of Bowel Obstruction on CT
Source: J Imaging Inform Med. 2024 Feb 22;37(4):1411–23. doi: 10.1007/s10278-024-01023-y (PMC11300723; doi:10.1007/s10278-024-01023-y)
Supplement: Supplementary file 1 — Supplementary file1 (DOCX 1158 KB) [file 10278_2024_1023_MOESM1_ESM.docx]

**Supplemental Information**

Figures showing statistical analyses of subsets of the gastrointestinal tract are provided separately for the “foregut” (longitude < 40), “midgut” (40 < longitude < 80), and “hindgut” (longitude > 80) in Figures S1, S2, and S3. Subsets are based on measured longitudes. ROC analysis could not be performed for longitude since only subsets were included. Please note that “fore/mid/hind-gut” terminology is used for convenience and varies slightly from embryologic definitions.

| 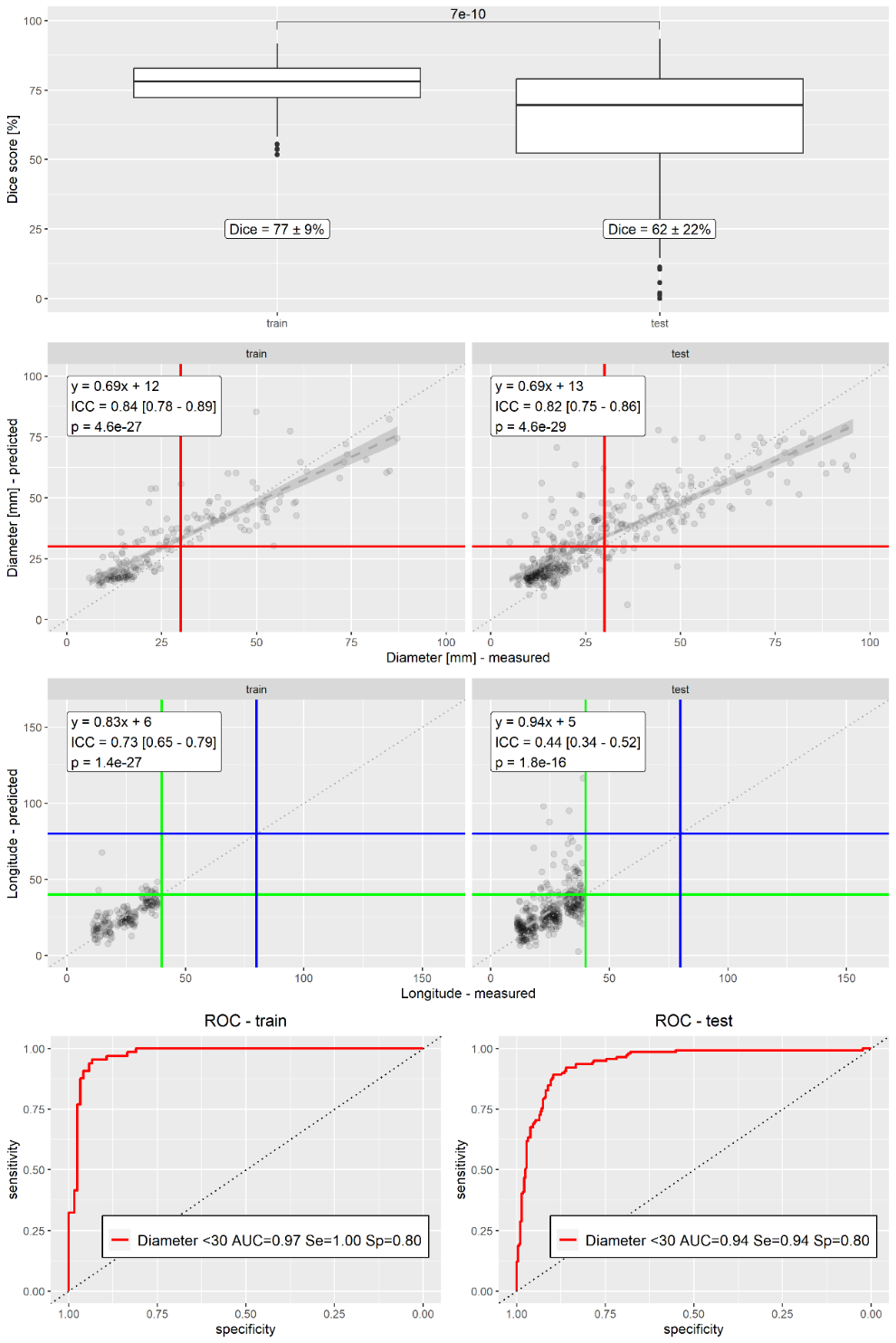 |
| --- |
| **Figure S1 – Statistical analysis of “foregut.”** |

| 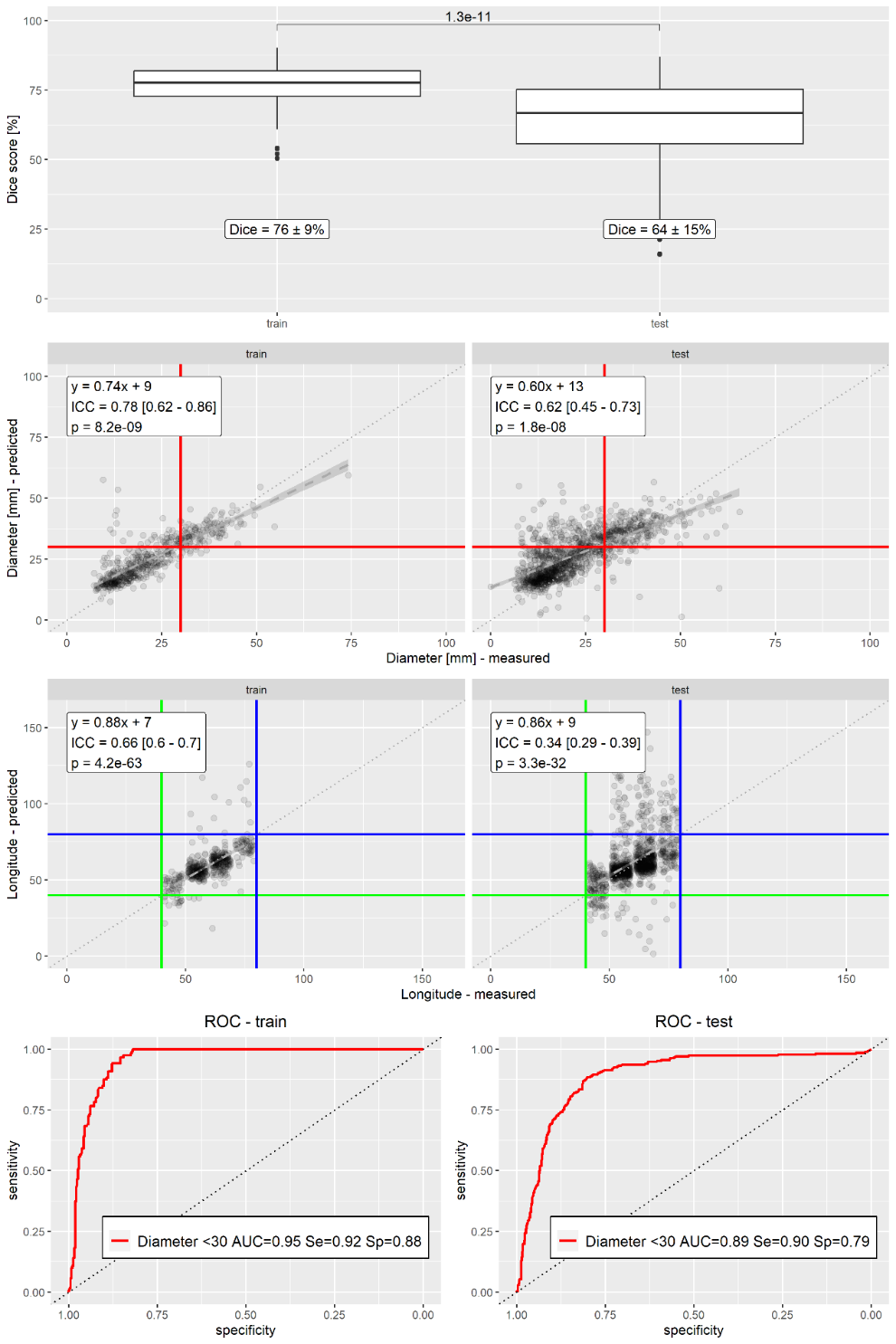 |
| --- |
| **Figure S2 – Statistical analysis of “midgut.”** |

| 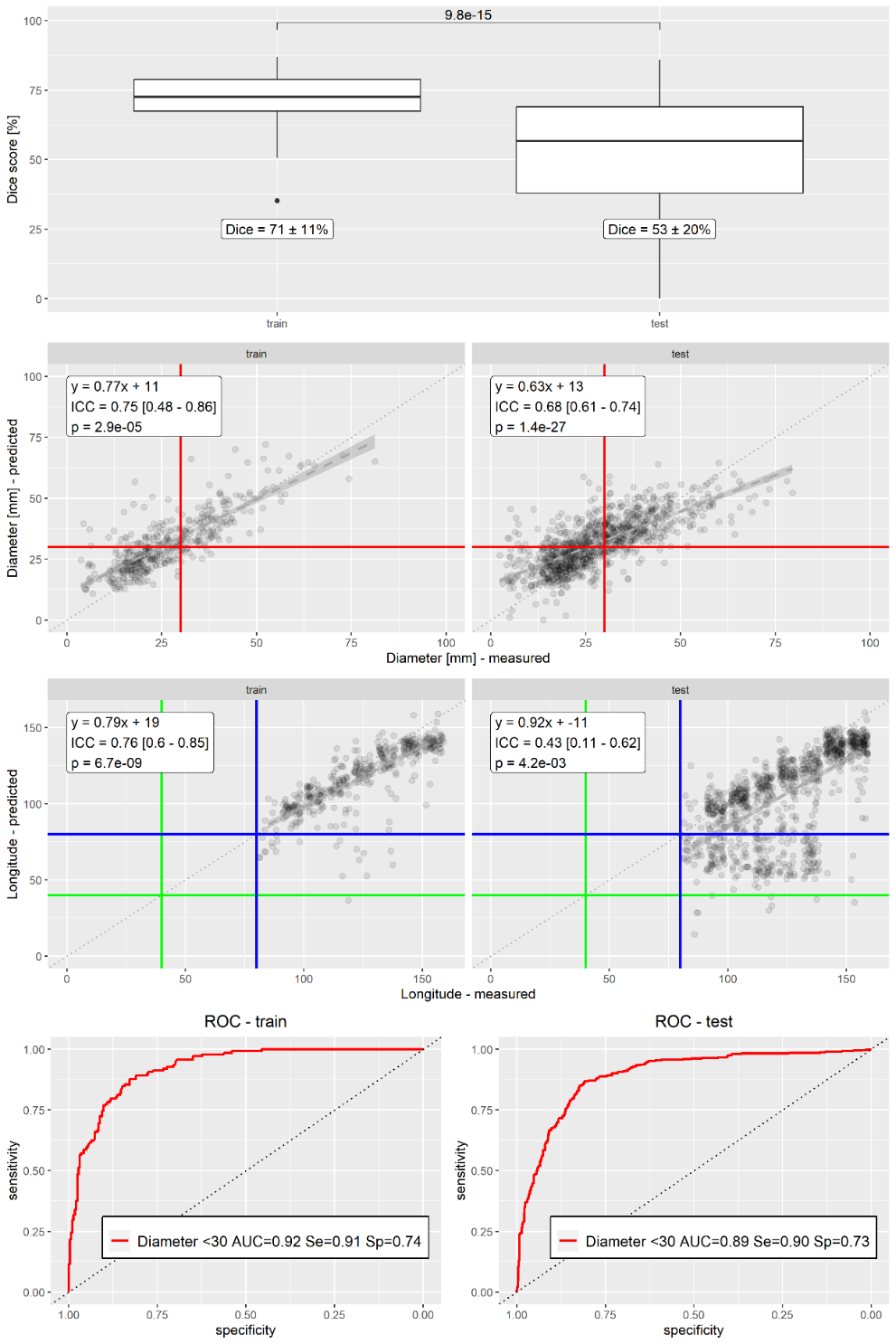 |
| --- |
| **Figure S3 – Statistical analysis of “hindgut.”** |
